# Identification of crosstalk genes relating to ECM‐receptor interaction genes in MASH and DN using bioinformatics and machine learning

**DOI:** 10.1111/jcmm.18156

**Published:** 2024-03-01

**Authors:** Chao Chen, Yuxi He, Ying Ni, Zhanming Tang, Wensheng Zhang

**Affiliations:** ^1^ Instrumentation and Service Center for Science and Technology Beijing Normal University Zhuhai China; ^2^ Pediatric Research Institute The Second Affiliated Hospital and Yuying Children's Hospital of Wenzhou Medical University Wenzhou China; ^3^ Zhuhai Branch of State Key Laboratory of Earth Surface Processes and Resource Ecology, Advanced Institute of Natural Sciences Beijing Normal University Zhuhai China; ^4^ Engineering Research Center of Natural Medicine, Ministry of Education, Advanced Institute of Natural Sciences Beijing Normal University Zhuhai China

**Keywords:** DN, ECM‐receptor interaction, immune infiltration, MASH, XGBoost

## Abstract

This study aimed to identify genes shared by metabolic dysfunction‐associated fatty liver disease (MASH) and diabetic nephropathy (DN) and the effect of extracellular matrix (ECM) receptor interaction genes on them. Datasets with MASH and DN were downloaded from the Gene Expression Omnibus (GEO) database. Pearson's coefficients assessed the correlation between ECM‐receptor interaction genes and cross talk genes. The coexpression network of co‐expression pairs (CP) genes was integrated with its protein–protein interaction (PPI) network, and machine learning was employed to identify essential disease‐representing genes. Finally, immuno‐penetration analysis was performed on the MASH and DN gene datasets using the CIBERSORT algorithm to evaluate the plausibility of these genes in diseases. We found 19 key CP genes. Fos proto‐oncogene (FOS), belonging to the IL‐17 signalling pathway, showed greater centrality PPI network; Hyaluronan Mediated Motility Receptor (HMMR), belonging to ECM‐receptor interaction genes, showed most critical in the co‐expression network map of 19 CP genes; Forkhead Box C1 (FOXC1), like FOS, showed a high ability to predict disease in XGBoost analysis. Further immune infiltration showed a clear positive correlation between FOS/FOXC1 and mast cells that secrete IL‐17 during inflammation. Combining the results of previous studies, we suggest a FOS/FOXC1/HMMR regulatory axis in MASH and DN may be associated with mast cells in the acting IL‐17 signalling pathway. Extracellular HMMR may regulate the IL‐17 pathway represented by FOS through the Mitogen‐Activated Protein Kinase 1 (ERK) or PI3K‐Akt–mTOR pathway. HMMR may serve as a signalling carrier between MASH and DN and could be targeted for therapeutic development.

## 1 INTRODUCTION

Non‐alcoholic fatty liver disease (NAFLD) is the most prevalent chronic liver disease worldwide and is closely related to type 2 diabetes.^1^ 20%–30% of patients with NAFLD progress to non‐alcoholic steatohepatitis (NASH).^2^ In early 2020, an international panel of experts led a consensus‐driven process to develop a more appropriate terminology for NAFLD. The term that was proposed was ‘metabolic dysfunction‐associated fatty liver disease,’ or ‘MAFLD’.^3^, ^4^ Referring to Lichtenstein's proposal,^5^ the notion of NASH is replaced by MASH in this paper. Some researchers even believe that MASH can be defined as ‘diabetic liver disease’.^5^ On the contrary, diabetic nephropathy (DN) is a significant healthcare challenge. It occurs in up to 50% of people with diabetes and is the leading cause of end‐stage renal disease (ESKD).^6^ Moreover, the evidence for an association and causal relationship between MAFLD and chronic kidney disease has been discussed in detail.^7^ Evidence suggests that besides the abnormal lipoprotein profile associated with metabolic syndrome and MAFLD, a range of cytokines and procoagulants secreted from the liver can affect the kidney.^8^ The accumulation of toxins in the body due to renal dysfunction has also been shown to exacerbate liver damage.^9^ For example, accumulated uric acid stimulates fructokinase activity and increases the sensitivity of hepatocytes to fructose metabolism, leading to liver fat deposition.^10^ Inevitably, these results suggested that these two diseases are related and can crosstalk each other as cytokines may function as a paradigm to reveal general crosstalk mechanisms and signalling networks.^11^, ^12^, ^13^ Studying cytokine signalling pathways will give more molecular mechanisms of crosstalk genes between MASH and DN.

MASH is characterized by hepatic steatosis, inflammation, hepatocellular damage and varying degrees of fibrosis. A central issue in this field is identifying those factors that trigger inflammation and thus drive the transition from MAFLD to MASH.^14^ Cellular molecules associated with MASH inflammation have been widely investigated. For example, Integrin β1 (ECM‐receptor interaction genes) enriching in extracellular vesicles mediates monocyte adhesion and promotes liver inflammation in murine MASH reported.^15^ Moreover, chemotaxin 2 (LECT2) promotes inflammation and insulin resistance in adipocytes via p38 MAPK pathways,^16^ and it plays as a hepatokine that links liver steatosis to inflammation via activating tissue macrophages in MASH.^17^ Several studies have also revealed the inflammatory response to MASH from an energetic perspective. For example, the inhibition of the mitochondrial citrate carrier, Slc25a1, reverts steatosis, glucose intolerance and inflammation in preclinical models of NAFLD/MASH.^18^

Inflammation in DN is also associated with intra‐ and extra‐cellular signalling. The crucial role of ECM protein accumulation in the development of DN has been well summarized.^19^ Recent studies demonstrated that some of the signalling pathways or molecules, including Notch,^20^ Wnt,^21^ mTOR,^22^ TLRs,^23^ NLRP3^24^ and small GTPase^25^ might play a pivotal role in the modulation of ECM regulation and expression in DN. In terms of energy metabolism, glucose induces IL‐1α‐dependent inflammation, and extracellular matrix proteins expression and deposition in renal tubular epithelial cells in DN was also reported.^26^

Signalling crosstalk is critical in informing diverse immune cellular decisions in inflammation development, triggering disease onset and progression. As in MASH, ECM‐receptor interaction genes, intracellular signalling pathways and energy fluxes affecting DN processes are related to cellular signalling communication. However, no clear advancement has been seen in how ECM‐receptor interaction genes can affect crosstalk genes in MASH and DN. We propose a hypothesis as a solution to address this problem. Since immunity and inflammation are common mechanisms in MASH and DN, the pathways enriched from the crosstalk genes of MASH and DN should contain immune pathways and could be influenced by ECM‐receptor interaction genes. Assuming that extracellular signals from ECM‐receptor interaction genes can stimulate cellular stress responses via crosstalk genes, such responses should include intracellular alterations in transcription factors involved in immunity and inflammation. Further, suppose such responses can be consistently concluded in terms of transcriptional expression, cellular differentiation, signalling pathways and even glucose metabolism. In this case, the association of these CP genes with the disease will be significantly enhanced. Therefore, studying the relationship between MASH and DN combined with ECM‐receptor interaction genes can contribute to understanding the pathogenesis mechanisms underlying their development and guide coordinated interdisciplinary management in clinical settings.

## 2 MATERIALS AND METHODS

### 2.1 Data downloading

We screened samples related to MASH and DN from the GEO (https://www.ncbi.nlm.nih.gov/geo) database and downloaded the data. The screening criteria were as follows: count value >50, organism parameters were human, experiment type of dataset was microarray and gene count >10,000. After screening, the datasets GSE164760, GSE89632 from MASH, and GSE96804, GSE30122 from DN were included in this experiment. All patient information is listed in the attached table (Table S1). After parsing the data with the GEOquery R package (version 2.66.0), samples unrelated to disease and controls were removed. MASH contained 123 samples, of which 93 showed positive results. Similarly, DN contained 105 samples, of which 60 tested positive. Next, the ComBat method in the sva R package (version 3.46.0) was used to adjust for bias across batches of samples (see Figure S1). The R version 4.2.1 was used in this work.

### 2.2 DEGs analysis

After log_2_‐transformation and batch correction, DEGs analysis was performed on the MASH and DN datasets using the limma package (version 3.54.0). Genes with | log_2_ (fold change)| > 0.6, padj <0.05 in MASH (Table S2) and DN (Table S3) were defined as DEGs. DEGs and preliminary clustering analysis were presented using volcano and heatmaps. R package included: ggplot2 (version 3.4.0), EnhancedVolcano (version 1.16.0), and Pheatmap (version 1.0.12) R package. The intersection of DEGs is shown with VennDiagram (version 1.7.3).

### 2.3 Functional enrichment analysis

Functional enrichment analysis of DEGs was performed using the clusterProfiler (version 4.6.0) R package. In Gene Ontology (GO) and Kyoto Encyclopedia of Genes and Genomes (KEGG) enrichment analysis, pvalueCutoff = 0.05 or qvalueCutoff = 0.05 were considered filter parameters after pAdjustMethod was set to ‘BH,’ respectively. The enrichment results for GO are shown in bar charts, and the analysis results for KEGG are shown in bubble charts.

### 2.4 Identification of crosstalk genes and their co‐expression in ECM‐receptor interaction genes

The shared DEGs from MASH and DN are suspected to be crosstalk genes associated with both diseases. ECM‐receptor interaction genes (hsa04512) are downloaded from the KEGG database with KEGGREST (version 1.38.0) R package. We used a heatmap to observe the difference in expression patterns of these ECM‐receptor interaction genes in MASH (or DN) compared with the control and then calculated their Pearson correlation coefficients with crosstalk genes. The Hmisc package (version 4.7.1) was used to calculate the correlation, where *p*‐values <0.05 and |*r*| > 0.4 were considered correlated. Finally, the results of the correlation analysis were plotted on a heat map using the corrplot R package (0.92). These gene pairs with co‐expression relationships present in MASH and DN are named co‐expression pairs (CP) genes (Table S4). The Cytoscape (version 3.9.1) plugin ClueGO (version 2.5.9) was used for analysing the relationship between essential genes (crosstalk genes and ECM‐receptor interaction genes) and the KEGG pathway. For KEGG term searches, a term with three genes or 4% of genes was considered to be enriched.

### 2.5 Protein–protein interaction (PPI) network analysis

We use Cytoscape to acquire and analyse interacting proteins. Using crosstalk and ECM‐receptor interacting genes as seeds, all retrieved interacting proteins from InnateDB (counts 1332), IntAct (counts 392), IMEx (counts 386) and MINT (counts 16) were placed into the STRING database (https://string‐db.org/) to reconstruct PPI network. After importing all the genes of the PPI network nodes into Cytoscape, the network was split into community subnetworks using the fast greedy algorithm with the clusterMaker2 plugin.^27^ The subnetworks are considered a gene cluster that could interfere with MASH or DN, so their hub genes are identified with cytoHubba.^28^ In the subsequent analysis, we isolated the subnetwork consisting of CP genes from community subnetworks to examine the shared genes between the hub and CP gene.

### 2.6 Identification of key genes

We used AUC (Area Under The Curve)‐ROC (Receiver Operating Characteristic) curves to assess the potential of candidate CP genes as potential biomarkers in MASH or DN. When using the pROC (version 1.18.0) R package, the direction of calculation of AUC (‘>’or ‘<’) leads to enantiomeric results. Therefore, we assumed the AUC value greater than 0.6 in both directions of calculation results as the key CP genes affecting the disease. In other words, these genes are either upregulated or downregulated in MASH and DN and can be dichotomized for disease.

We then constructed a correlation network of key genes (|*r*| > 0.4).^29^ This graph is a subset of the CP gene correlation network, and the screening criterion was an AUC >0.6. To further determine which of these key CP genes are critical features of the disease, we evaluated the importance score of each gene using the machine learning method XGBoost package (version 1.6.0.1).^30^ In machine learning, 70% was used as the training dataset and 30% as the independent test dataset. The model was trained by a k‐fold cross‐validation method (*k* = 5). The lowest Binary classification error rate (error) was used as the evaluation criterion to determine the best‐estimated model. The top five genes' feature scores were given, and the model's accuracy for disease determination was also evaluated. Since the genes most representative of the disease were significantly correlated with those in the ECM‐receptor interaction genes, we examined the statistical information on their correlation using the corrplot R package.

### 2.7 Key CP genes functional enrichment analysis

To better understand the functions of all key CP genes and their possible interaction mechanisms. We enriched the key CP genes for KEGG functional analysis and then imported them into the STRING database to obtain the PPI network. Since this PPI network was insufficient to characterize a significant correlation between genes, we used Adobe Illustrator soft to complement the correlation edges between genes based on our results. Not only that, but we also supplied additional information on genes in pathway annotation that we considered necessary.

### 2.8 Immune infiltration analysis

CIBERSORT is a versatile computational method for quantifying cell fractions from bulk tissue gene expression profiles.^31^ We used CIBERSORT to obtain immune infiltration matrices from the gene expression datasets of MASH and DN. Heatmap and ggplot2 R packages were used to visualize the computational results, and the corrplot R package was used to describe the correlation between key CP genes and immune cells.

## 3 RESULTS

### 3.1 Identification and enrichment analysis of DEGs in MASH and DN

**FIGURE 1:**
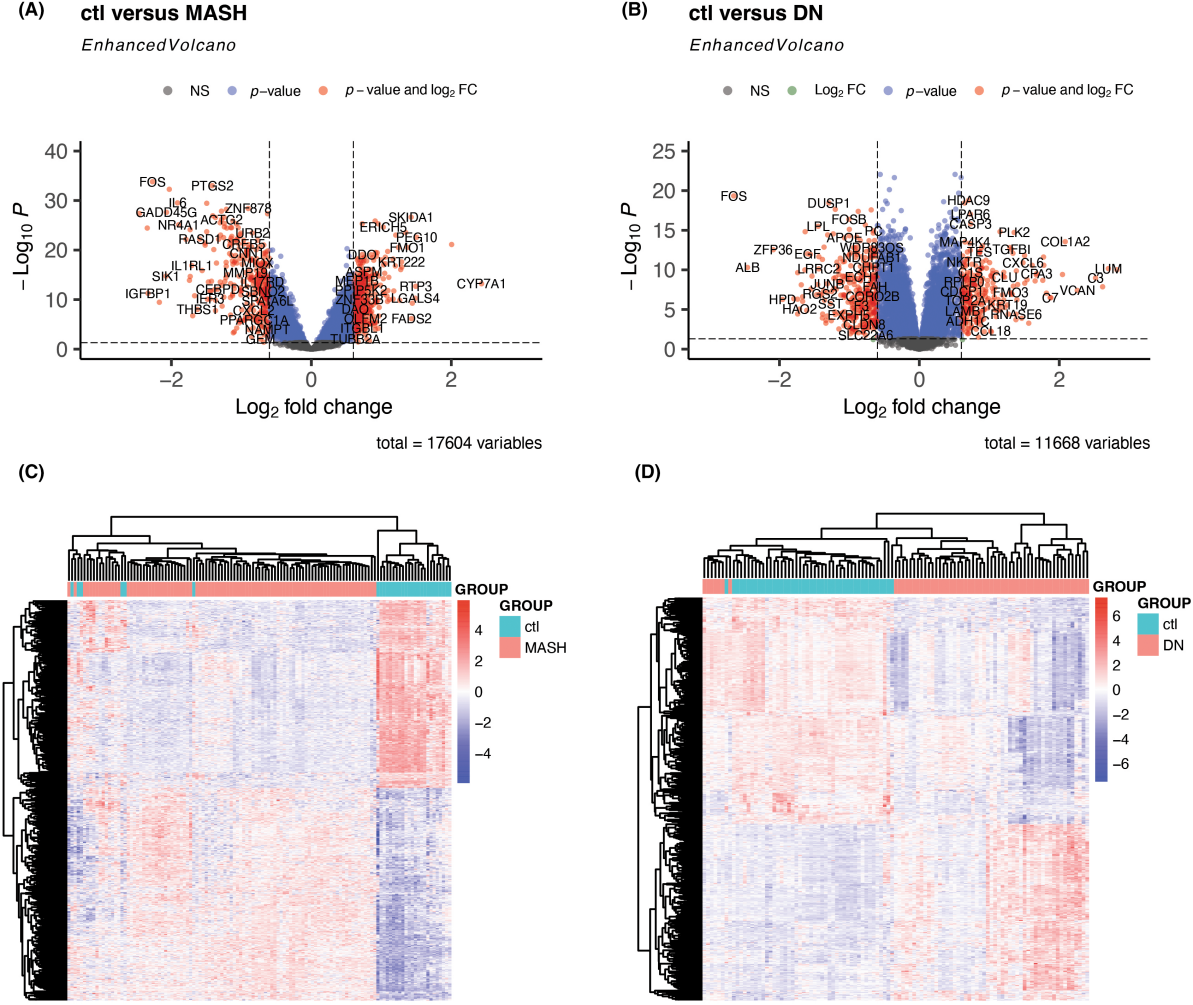
Volcano map and heatmap of DEGs in MASH and DN. (A and B) Volcano map of MASH and DN genes datasets. | log2 (fold change) | > 0.6, padj <0.05; (C and D) Heatmap of MASH and DN genes datasets. Each row represents a gene, and the colour represents the expression level. The colour bar in the upper right corner of the figure is used as a reference for gene expression levels.

The strategy of bioinformatics analysis is performed as shown in Figure S2. We collected biochip data related to MASH and DN from the GEO database. Then after eliminating batch effects and other unwanted variables, two datasets were gathered. The MASH datasets (GSE164760 and GSE89632) contain 30 control and 93 case samples. The DN datasets (GSE96804 and GSE30122) contain 45 control and 60 case samples. In DEGs analysis, we found that FOS, which can form a complex with JUN/AP‐1 transcription factor, was significantly downregulated in the MASH and DN datasets (Figure 1A,B). However, similar genes with significant Fold change values and small *p*‐value are not easily found in the upregulated genes. Therefore, we made a heatmap to examine the clustering of DEGs in different diseases (Figure 1C,D) and further performed GO analysis for DEGs in a single disease.

**FIGURE 2:**
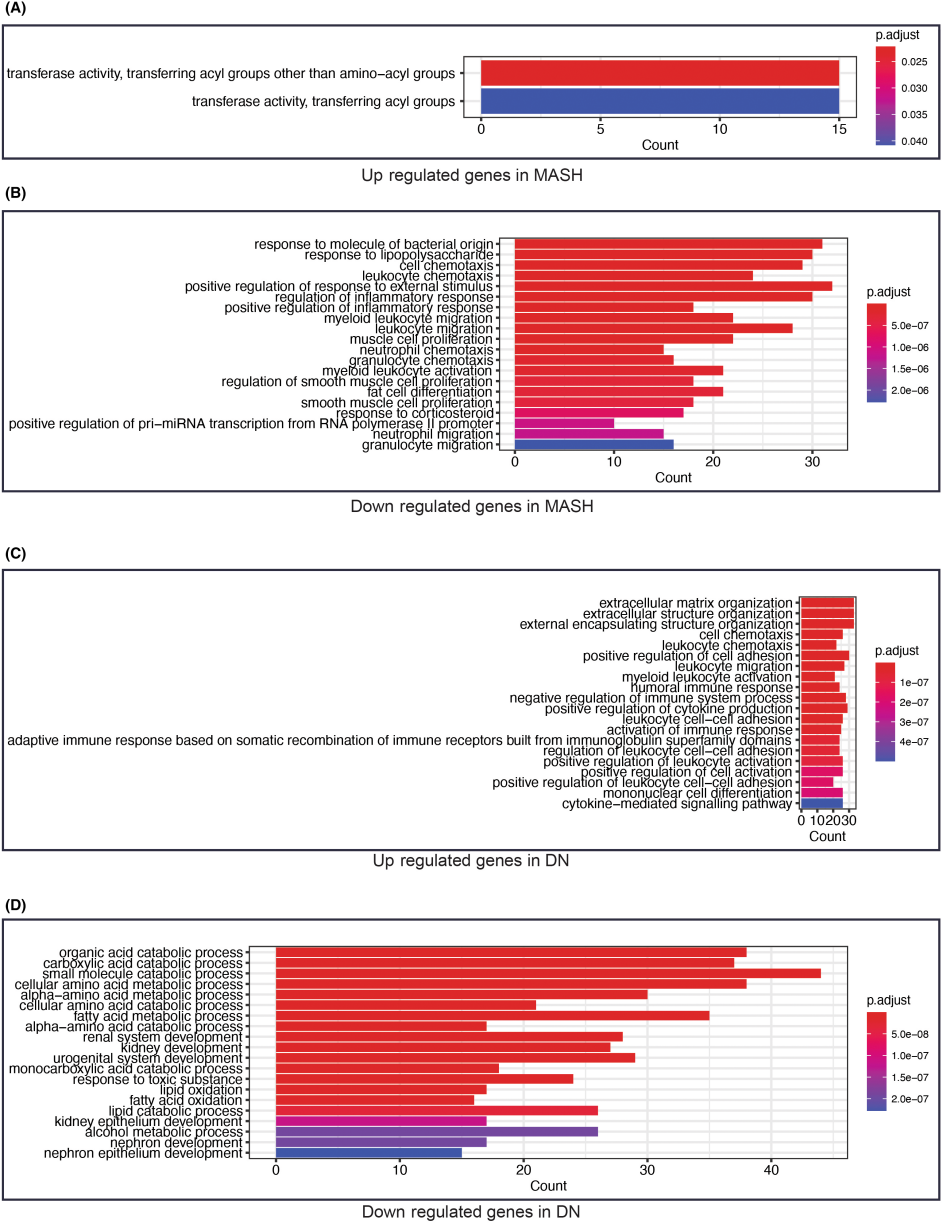
Go enrichment analysis of DEGs. GO hierarchy contains three sub‐ontologies (BP, MF and CC). The statistical method is BH (Benjamini and Hochberg) correction, and the top 20 terms were shown in the barplot.

We found that MASH‐upregulated genes were only enriched in the pathway associated with transferring acyl (Figure 2A). Conversely, the GO annotation results of DN upregulated genes were more abundant, including pathways related to the extracellular matrix, immune, cytokine‐related pathways, et al. (Figure 2C). For downregulated genes, MASH‐related genes were enriched in inflammatory response (Figure 2B), cell migration and proliferation. On the contrary, genes related to DN were enriched in lipid oxidation and amino acid metabolism (Figure 2D). In brief, the heatmap and GO enrichment analysis show that the expression patterns of DEGs were not the same in MASH and DN, but they all have the FOS gene downregulated. FOS‐involved AP‐1 transcription factor is acknowledged as a master integrator of a myriad of extracellular signals allowing cells to adapt to changes in their environment.^32^ This result implies that we can further investigate the various mechanisms of extracellular matrix stimulation of MASH and DN's development through FOS.

### 3.2 Identification and enrichment analysis of crosstalk genes

**FIGURE 3:**
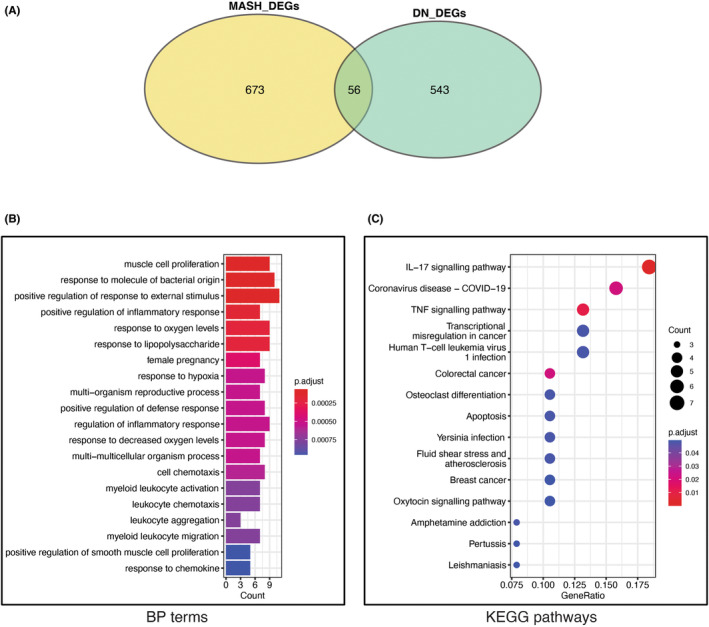
Enrichment analysis of crosstalk genes. (A) Venn diagram of the intersection of MASH and DN genes; (B) TOP20 GO BP terms of crosstalk genes; (C) TOP20 KEGG pathways of crosstalk genes.

To determine whether MASH and DN are similar in disease progression, we collected the common part of the DEGs and performed pathway enrichment analysis on them. Fifty‐six genes were altered in both MASH and DN (Figure 3A). Biological Process (BP) enrichment results showed that the crosstalk DEGs from MASH and DN were enriched in cell proliferation, inflammatory response, response to oxygen, cell chemotaxis and leukocyte migration (Figure 3B). The significant pathways in KEGG enrichment contain IL‐17, Tumour Necrosis Factor (TNF) signalling pathway, apoptosis, cancer, oxytocin signalling pathway, fluid shear stress and atherosclerosis (Figure 3C). These data suggest improper inflammatory and immune responses are present in both MASH and DN. Equally important, as IL‐17‐driven signalling drives several effector functions, including chemokine induction, cellular infiltration and tissue barrier function and remodelling,^33^ it is unsurprising that IL‐17 could mediate ECM production.^34^ Thus, we further confirmed whether the ECM‐receptor interaction genes influence MASH and DN processes is possible, although none of the 56 differential genes belong to ECM‐receptor interaction genes.

### 3.3 Correlation and KEGG analysis of crosstalk genes with ECM‐receptor interaction genes

To further clarify whether ECM‐receptor interaction genes are DE in the disease, we investigated the heatmap of this fraction of genes in MASH and DN. The results showed differences between the control and disease‐causing groups (Figure S3A,B). Even though some ECM‐receptor interaction genes could not pass the screening criteria in DEGs analysis, we included them in the gene set for calculating the correlation coefficient with the crosstalk genes (Figure S3C,D). We proposed that co‐expressed gene pairs that can appear in both MASH and DN may be essential for ECM to link disease progression. Hence, we defined these 171 gene pairs (Table S4), with co‐expression correlation coefficients greater than 0.4 and appearing in both MASH and DN, as CP genes.

**FIGURE 4:**
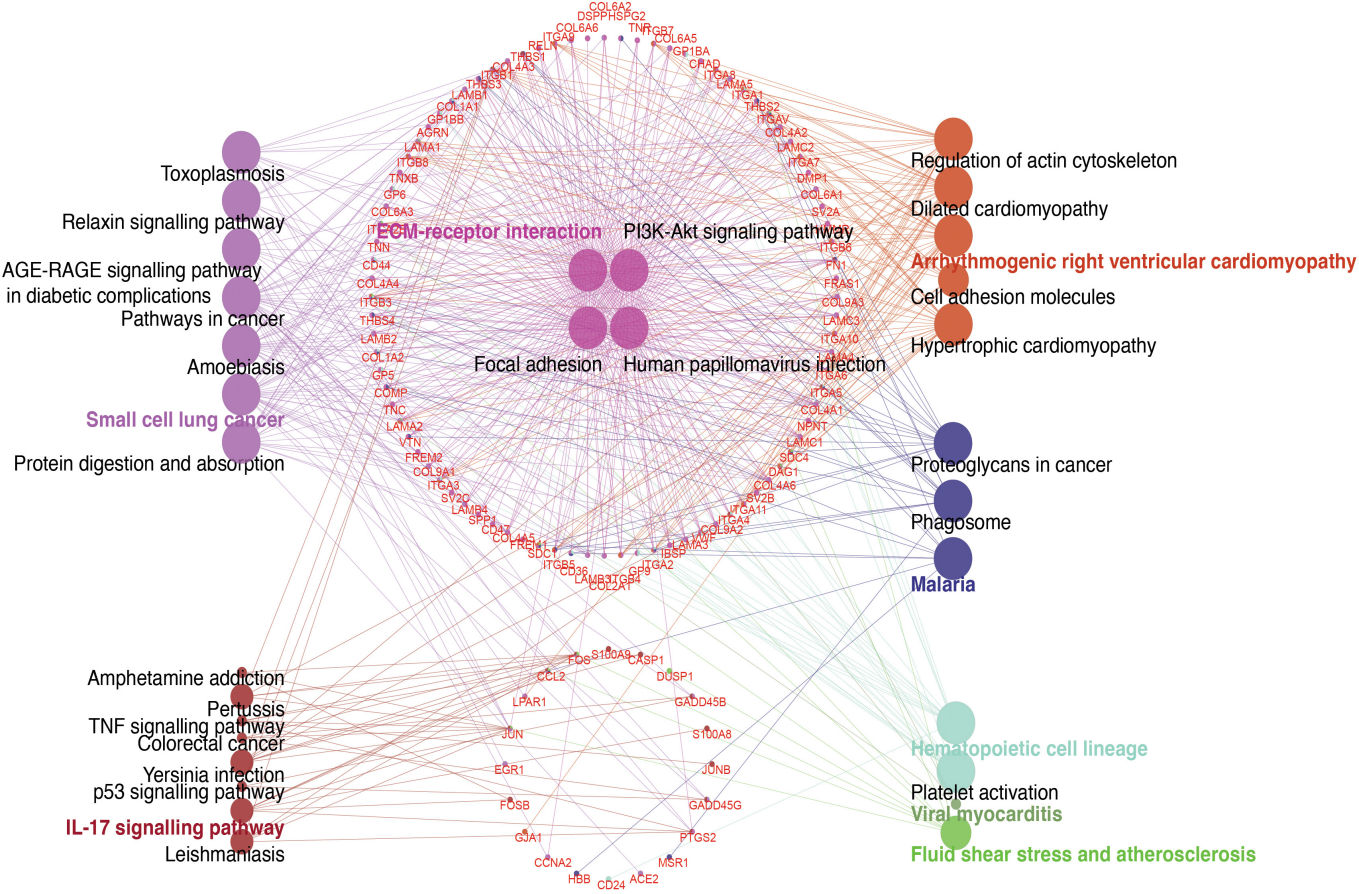
KEGG analysis of crosstalk genes and ECM‐receptor interaction genes. The red font in the circled diagram indicates genes flanked by KEGG pathways.

**FIGURE 5:**
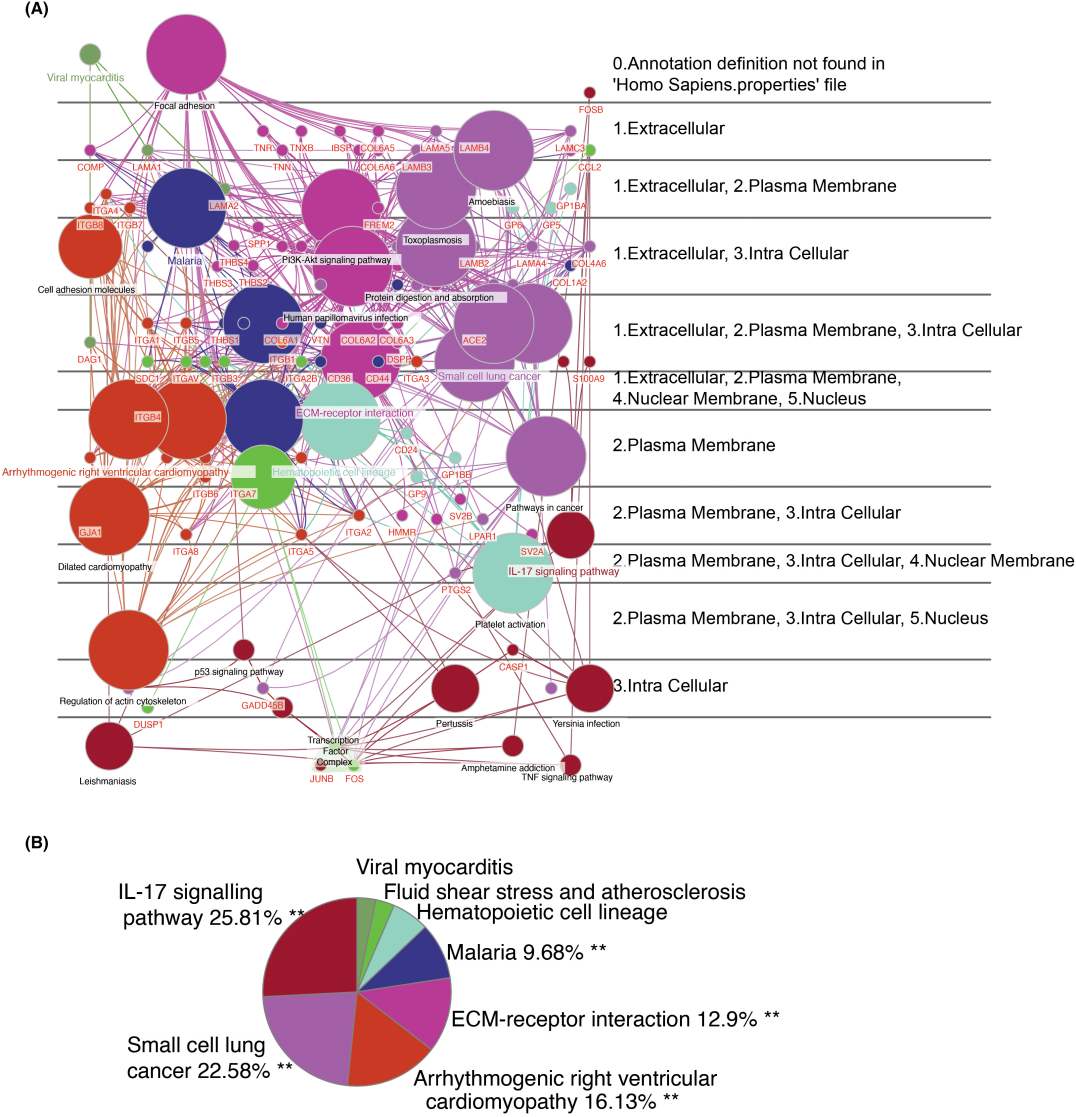
Distribution map analysis of crosstalk genes and ECM‐receptor interaction genes. (A) Cerebral plot of the network with ClueGO terms/pathways; (B) Pie plot of ClueGO terms/pathways. ** *p*‐value < 0.01.

In addition, to explore how ECM‐receptor interaction genes affect disease progression in MASH and DN through crosstalk genes, we used the KEGG in ClueGO for pathway analysis. We found that ECM‐receptor interaction genes are highly analogous with other KEGG pathways, such as the PI3K‐Akt signalling pathway (Figure 4). Nevertheless, ECM‐receptor interaction genes can be cotargeted with crosstalking genes on cancer, IL‐17, cardiomyopathy, atherosclerosis and other KEGG pathways. Interestingly, we found that AGE‐RAGE signalling pathways in diabetic complications were also enriched, which is consistent with the fact that diabetes and MASH interact with each other.^35^ In addition, by identifying the gene distribution map in the cell (Figure 5A), we found that JunB Proto‐Oncogene (JUNB) and FOS transcription factors in the nucleus can attach to the extracellular matrix through the TNF signalling pathway, IL‐17 pathway and ECM‐receptor interaction genes. Furthermore, bridge genes in these linkages, such as Secreted Phosphoprotein 1 (SPP1, localized outside the cell) and HMMR (localized on the membrane), may be able to shuttle freely within and outside the membrane. Finally, the results of the statistical plots of the relevant terms indicate that the pathways through which ECM genes affect crosstalk genes may be multifaceted, including IL‐17, cancer, cardiomyopathy and ECM‐receptor interaction genes themselves (Figure 5B).

### 3.4 Construction of PPI network and analysis of possible gene functional clusters

**FIGURE 6:**
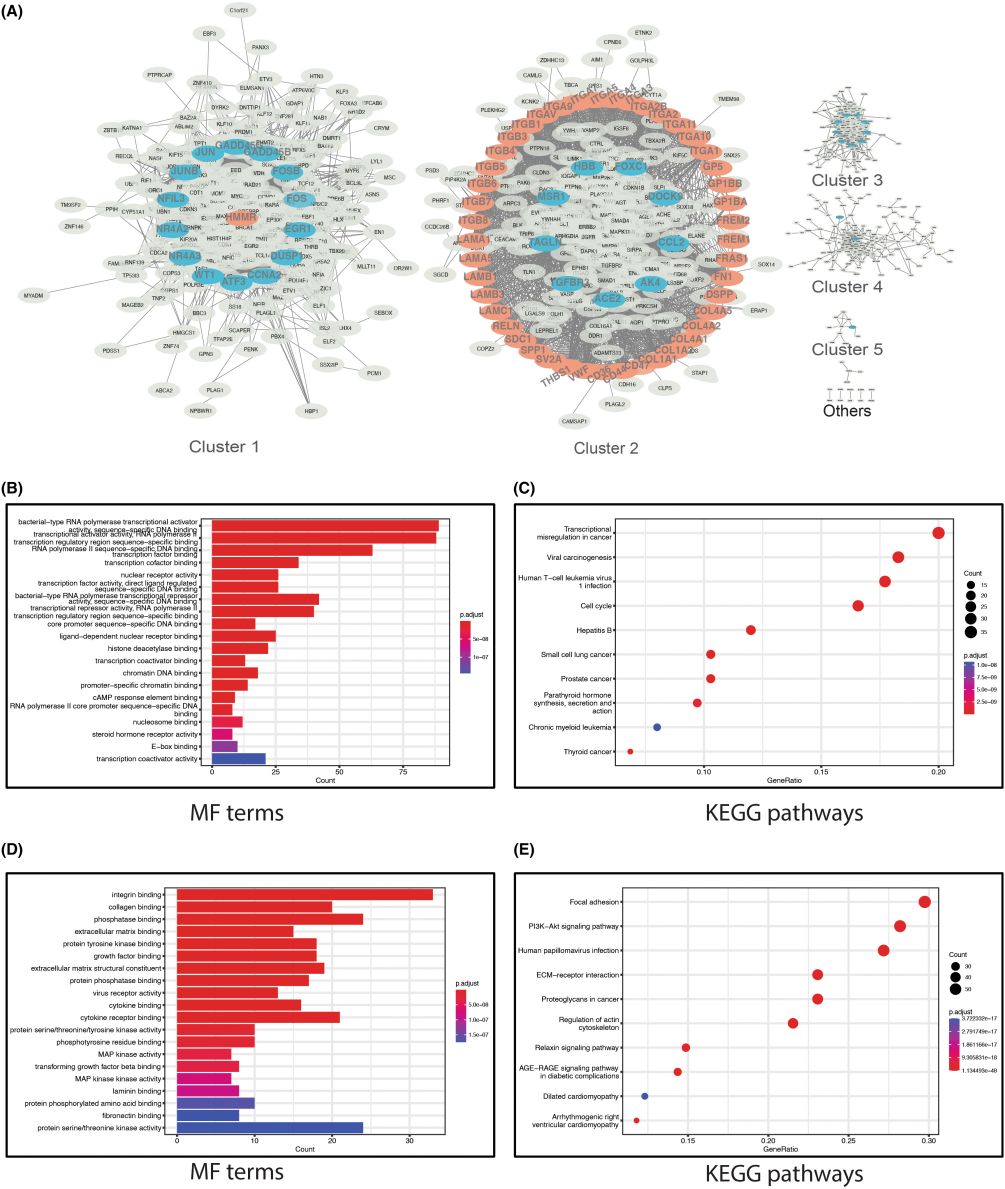
Construction of PPI subnetwork and function enrichment analysis. (A) PPI network communities analysis, Crosstalk genes are shown in blue, ECM‐receptor interaction genes are shown in orange; (B) MF enrichment analysis of Cluster 1; (C) KEGG enrichment analysis of Cluster 1; (D) MF enrichment analysis of Cluster 2; (E) KEGG enrichment analysis of Cluster 2.

Since exosomal mRNAs are functional, recipient cells can take them up and translate them^36^; under the intracellular phase separation mechanism, mRNA‐protein can bind and determine the spatial binding pattern of specific intracellular droplets.^37^ The ID‐transformed ECM‐receptor interaction genes and crosstalk genes were used as seeds (Table S5) in Cytoscape to search for the interaction proteins. Then, the connections between the proteins were obtained at the STRING URL and put back into Cytoscape to construct the PPI network. The complex network includes 880 nodes and 15,865 edges (Table S6). Using the fast greedy algorithm, we split the genes in the complex network into six major subclusters (Figure 6A). Among them, cluster 1 and cluster 2 covered genes from ECM‐receptor interaction and crosstalk genes. Therefore, we define these two clusters as critical clusters related to ECM‐receptor interaction genes.

Detecting communities allows us better to understand network dynamic processes' properties.^38^ Therefore, we performed GO molecular function (MF) and KEGG enrichment analysis on the genes of clusters 1 and 2. MF results revealed that cluster 1 was mainly related to nucleic acid binding (Figure 6B), while cluster 2 was related to MAPK kinase activity, cytokine receptor and growth factor binding (Figure 6D). Further, KEGG enrichment analysis revealed that cluster 1 is mainly involved in transcriptional misregulation in cancer, cell cycle (Figure 6C), and cluster 2 was enriched in ECM‐receptor interaction genes, PI3K‐Akt, Focal adhesion, AGE‐RAGE signalling pathway in diabetic complications (Figure 6E).

### 3.5 Identification of key CP genes

**FIGURE 7:**
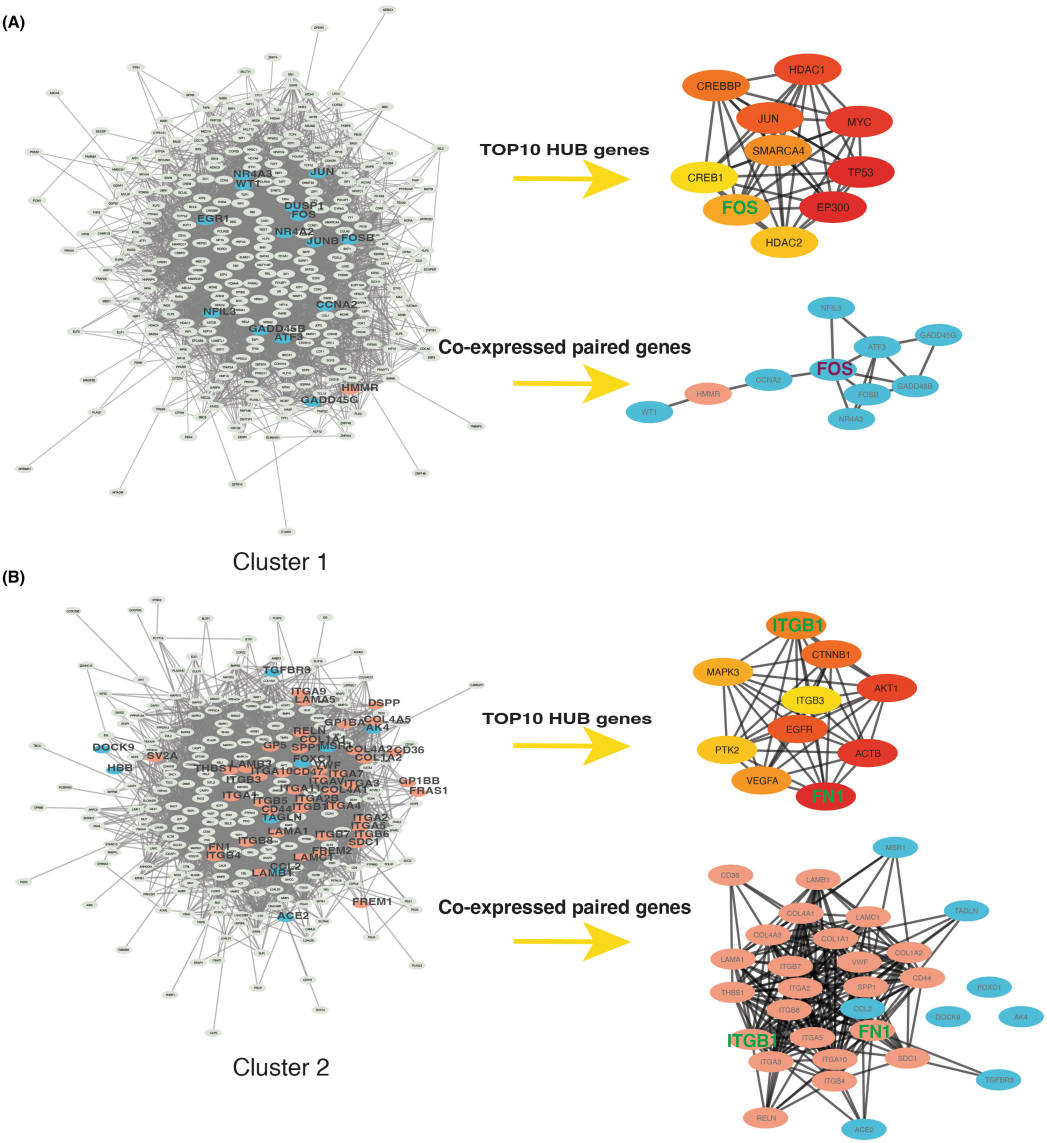
Hub and CP genes in subcluster. (A) Hub and CP genes in Cluster 1; (B) Hub and CP genes in Cluster 2; The yellow to red indicates the hub gene. Crosstalk genes are shown in blue, and ECM‐receptor interaction genes are shown in orange.

We want to clarify whether the hub genes of a PPI network are present in our isolated CP genes. First, we detect 10 hub genes of the respective networks in Cluster 1 and 2. Then, the CP genes in the clusters were constructed as separate subnetworks. In cluster 1, the hub gene FOS is shared in the PPI subnetwork of CP genes (Figure 7A). In cluster 2, the hub genes Integrin Subunit Beta 1(ITGB1) and Fibronectin 1 (FN1) are shared in the PPI subnetwork of CP genes (Figure 7B). In both key clusters, the PPI network of CP genes contained a fraction of the hub genes in the PPI network. Although FOS, ITGB1 and FN1 are easily understood as key core genes related to MASH and DN, they have some uncertainties. For instance, STRING is not always experimentally validated when constructing PPI networks, and the algorithm is somewhat random when discovering community subnetworks of PPI networks. Therefore, which candidate CP genes significantly impact MASH and DN needs further analysis.

**FIGURE 8:**
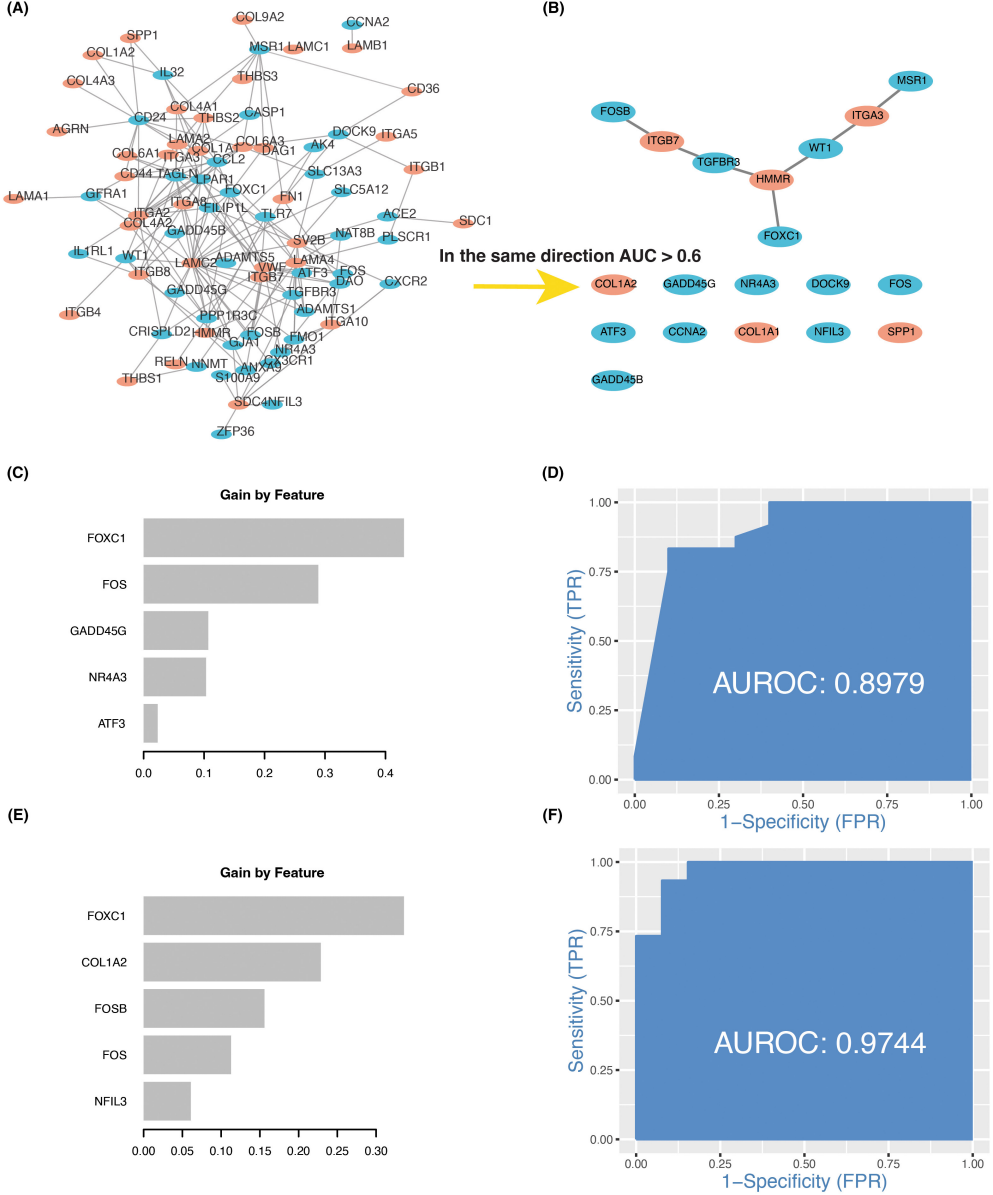
The coexpression network of CP genes and their features in XGBoost. (A) The coexpression network of CP genes; (B) CP gene of AUC >0.6; (C) Top five genes of feature's importance score in MASH; (D) Overall prediction score of XGBoost model in MASH; (E) Top five genes of feature's importance score in DN; (F) overall prediction score of XGBoost model in DN.

ROC is one of the essential evaluation metrics to check the performance of a binary classification model. Therefore, we used AUC values to assess which candidate CP genes were associated with MASH and DN (Table S7, Figure S4). By assessing the ability of genes to classify diseases, we listed 19 key CP genes in the table (Table S8). The co‐expression relationship network of these genes is shown in Figure 8A,B.

### 3.6 Machine learning–XGBoost analysis of key CP genes

**FIGURE 9:**
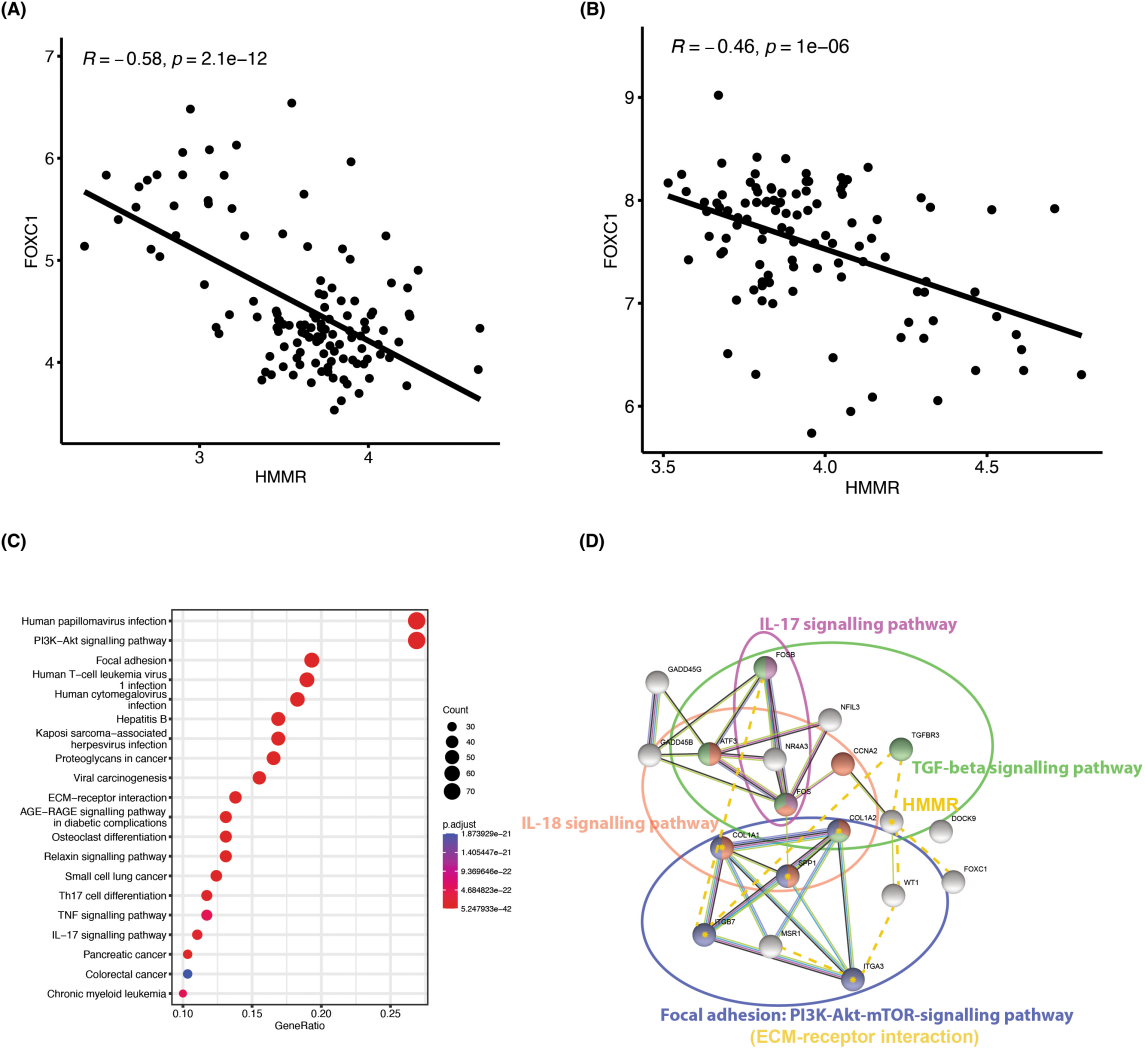
Functional pathways of key CP genes. (A) Analysis of correlation between FOXC1 and HMMR in MASH; (B) Analysis of correlation between FOXC1 and HMMR in DN; (C) KEGG‐enrichment analysis of key CP genes; (D) PPI network of key CP genes. Yellow star‐marked orbs represent ECM‐receptor interacting genes.

To identify the critical genes from CP genes by which the MASH and DN could be predicted, we use an efficient gradient boosting (XGBoost) model to screen critical genes (Figure 8C,E). Compared to the simple random forest and ridge regression, XGBoost has both strengths of regularization (ridge) and decision trees (random forest).^39^ Previous studies have shown superior performance of XGBoost in MASH and DN disease prediction.^40^, ^41^, ^42^ The XGBoost model has a variable importance calculation, which summarizes the degree of influence of the feature variables in the training process to predict disease based on the parameter gain of a feature variable during the prediction process.^43^ In this work, estimated by 5‐fold cross‐validation, the optimized XGBoost classifier can achieve a higher mean AUC of 0.89 at the overall level (Figure 8D,F). In addition to FOS, FOXC1 is considered a significant factor in classifying MASH and DN in our work. FOXC1, as a transcription factor, is involved in normal embryonic development, regulates the development and function of many organs, and plays a crucial role in tumour development and metastasis.^44^ Figure 9A,B show that the HMMR co‐expressed with the FOXC1. It is suggested that the community communication of CP genes and crosstalk genes may partially depend on the HMMR‐FOXC1, even though this expression relationship is negatively correlated.

### 3.7 Enrichment analysis of functional pathways in the PPI network for CP genes

The KEGG enrichment analysis suggested that the PI3K−Akt signalling pathway, Focal adhesion, ECM−receptor interaction, AGE‐RAGE signalling pathway in diabetic complication, TNF signalling pathway and IL−17 signalling pathway were enriched in19 key CP genes (Figure 9C). These 19 key CP genes were constructed into a PPI network using the STRING database to understand key genes' function better. The co‐expression relationships between them were represented with dashed lines (Figure 9D). Nineteen key CP genes were associated with four signalling pathways: the signalling pathways related to cytokines IL‐17 and IL‐18, the TGF signalling pathway related to inflammation, and the PI3K/AKT/mTOR pathway about energy. It is important to emphasize that the PI3K‐Akt–mTOR pathway genes in the network, together with HMMR, can be classified as ECM receptor interaction genes. Evidently, these 19 genes can be divided into two modules: cytokine and immune pathways represented by FOS transcription factors and mTOR signalling pathways that can be secreted outside the cell and sense energy changes described by secreted phosphoprotein SPP1. These two types of subnetworks are tightly linked through the IL‐18 signalling pathway. In the network constructed with the dotted line, HMMR, the ECM‐receptor interaction, is not only the hub gene but also seems to be linked to both of the above modules through some bridging genes.

### 3.8 Immune infiltration analysis

**FIGURE 10:**
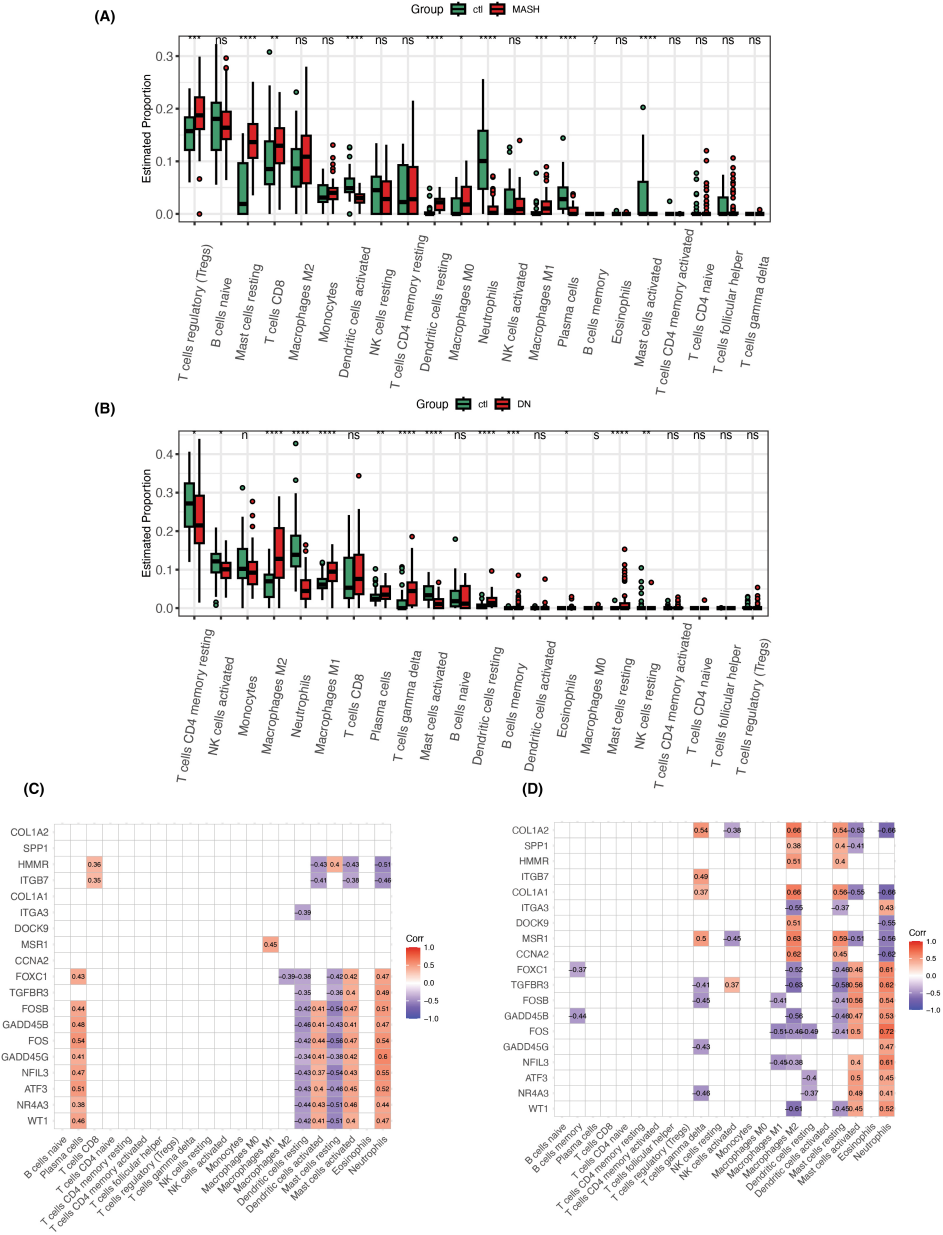
Immune infiltration. (A) Composition of immune cells in MASH; (B) Composition of immune cells in DN; (C) Quantitative differences in the composition of immune cells in MASH; (D) Quantitative differences in the composition of immune cells in DN. *p‐value <0.05, **p‐value <0.01, ***p‐value <0.001, ****p‐value <0.0001.

Since HMMR has been associated with immune infiltration in many experiments,^45^ studying transcription factors co‐expressed with it might be able to infer the connection from the cell surface to the nucleus. FOXC1 and WT1 Transcription Factor (WT1)^46^ are transcription factors among the three co‐expressed genes, while TGFBR3^47^ is a growth factor receptor. Furthermore, it is well known that the transcription factors such as AP‐1 are important regulators of the immune response.^48^ Therefore, studying the relationship between FOXC1 and immune infiltration also becomes attractive. We consequently used the CIBERSORT algorithm to analyse the immune infiltration in the MASH and DN datasets. These findings suggest that MASH and DN differed significantly in the types of immune cells. After a detailed comparison, we found that Mast cells resting in MASH, and Macrophages M2 in DN are easily used to classify the types of immune cells (Figure 10A,B). Moreover, when mast cells activated were downregulated, mast cells resting were upregulated in MASH and DN. More interestingly, mast cells that can be a source of IL‐17^49^ are significantly associated with perturbations of transcription factors such as FOS, FOXC1 and WT1(Figure 10C,D).

## 4 DISCUSSION

The cytokine environment and immune cells trigger a series of events called inflammatory processes. Many cytokine genes are influenced by the cytokines regulated by AP‐1,^32^ indicating that AP‐1 allows cells to respond to different extracellular signals. AP‐1 is a dimeric transcription factor composed of proteins belonging to the Fos (c‐Fos, FosB, Fra‐1 and Fra‐2), Activating Transcription Factor (ATF), Jun Proto‐Oncogene (Jun) and MAF BZIP Transcription Factor MAF multigene families.^48^ In our DEGs analysis, FOS showed a significant downregulation trend in MASH and DN (Figure 1A,B). Not only that, among the key CP genes shown in Figure 9D, FOSB and ATF3 were also downregulated in diseases (Tables S2 and S3). Considering the inflammatory response of tissues is subject to the involvement of ECM,^50^, ^51^, ^52^ the potential role of ECM‐receptor interaction genes and their relationship with FOS in MASH and DN is worth exploring.

The previous studies on the IL‐17 signalling pathway have indicated that metabolic inflammation‐associated IL‐17A leads to MASH^53^ and that the IL‐17 signalling pathways are a potential therapeutic target for rapidly progressive glomerulonephritis.^54^ In our work, the KEGG enrichment analysis identified that the most significant enrichment pathway for crosstalk genes is the IL‐17 signalling pathway, partially overlapping with the Apoptosis and TNF‐signalling pathway associated with inflammation/immunity (Figure 3). In addition, several ECM‐receptor interaction genes were co‐expressed with crosstalk genes in MASH or DN (Figure S3). This result suggests that co‐expression relationships at the gene level may have biological implications on the progression of these diseases. We were also particularly interested in whether ECM‐receptor interaction genes can disturb crosstalk genes by joining in the same KEGG pathway (such as the IL‐17 signalling pathway) between MASH and DN. In Figure 5A, crosstalk genes and ECM‐receptor interaction genes can form a dense crossover in KEGG terms. The transcription factors represented by JUNB (also of AP‐1) and FOS can associate with the proteins on the extracellular plasm membrane through several KEGG terms. Our results suggest extracellular or membrane ECM‐receptor interaction genes (e.g. Secreted Phosphoprotein 1(SPP1) and HMMR) may maintain communication with the IL‐17 signalling pathway (such as JUNB and FOS) through some shared KEGG pathway, which facilitates the activation of cellular AP‐1 transcription factors by cytokines from blood.

In addition to the overlap of KEGG terms, the PPI network can also suggest communication between two gene sets. In this work, the crosstalk genes and ECM‐receptor interaction genes remain linked when the complex network is split into smaller community networks (Figure 6). Cluster 1 seems related to transcription, while Cluster 2 relates to PI3K‐Akt, ECM‐receptor interaction genes and AGE‐RAGE signalling pathways in diabetic complications. This result suggests that linkages between ECM‐receptor interaction genes or crosstalk genes are concentrated in several KEGG pathways mentioned above. Furthermore, Figure 7 shows that the two clusters of hub genes are FOS (from crosstalk genes), FN1 and ITGB1 (from ECM‐receptor interaction genes), respectively. The occurrence of these three hub genes in CP genes suggests that hub genes may play a decisive role in specific biological processes and establish a direct link between ECM‐receptor interaction genes and crosstalks through the PPI network.

Since ECM‐receptor interaction genes are not among the 56 DEGs shared between MASH and DN, it is necessary to remove those CP genes that cannot accurately classify the positive samples from two diseases with ROC curves (Figure S4). Screened by AUC values, nineteen CP genes that could affect communication between ECM‐receptor interaction genes and 56 crosstalk genes were identified (Figure 8A,B). HMMR occupied the focus position in the co‐expression network from 19 CP genes. Furthermore, although the FOS gene has been identified as the hub gene of PPI cluster 1 (Figure 7A), crosstalk genes more representative of MASH and DN need further confirmation. In recent years, machine learning methods have been applied to various fields of bioinformatics. For example, Wang et al. developed a prediction model called DMFGAM for predicting hERG blockers,^55^ and A graph convolutional neural (GCN)‐based network and conditional random field (CRF) method, GCNCRF, to predict human lncRNA‐miRNA interactions.^56^ Sun et al. proposed a new deep‐learning algorithm, GCNAT, for predicting potential associations of disease‐related metabolites.^57^ The XGBoost is also more widely used in the medical field.^58^, ^59^, ^60^ XGBoost, for Extreme Gradient Boosting, is a scalable, distributed gradient‐boosted decision tree (GBDT) machine learning algorithm. While the XGBoost model typically achieves higher accuracy than a single decision tree, it sacrifices the intrinsic interpretability of the decision tree. In this work, by applying the XGBoost machine learning approach, we identified that, besides FOS, FOXC1 is also a critical transcription factor in MASH and DN (Figure 8C,E). Moreover, these two transcription factors showed good categorization ability with AUC = 0.90 in MASH and AUC = 0.97 in DN (Figure 8D,F), suggesting that FOS and FOXC1 play essential roles in the pathogenesis of MASH and DN.

**FIGURE 11:**
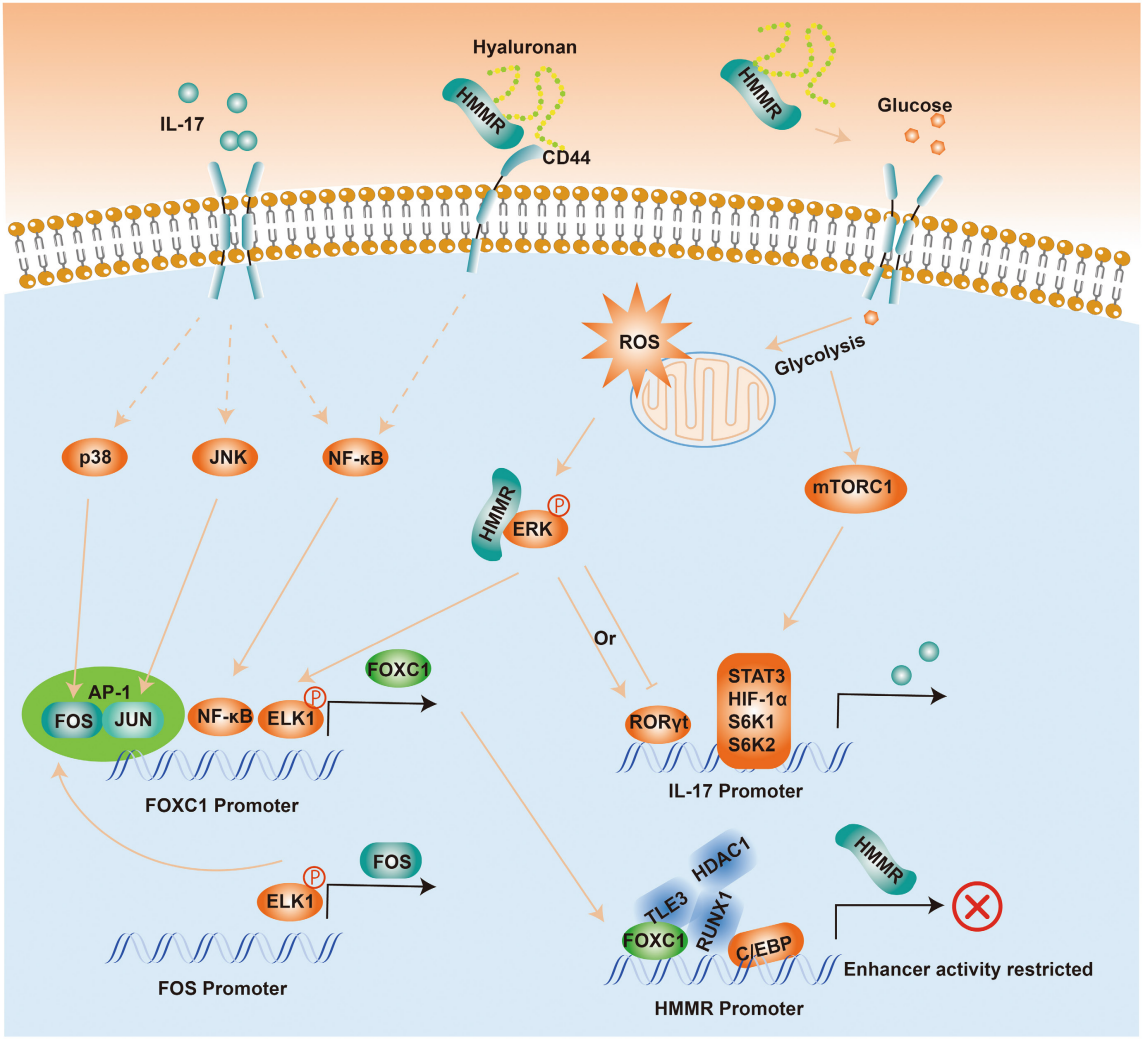
Relationship between FOS/FOXC1/HMMR and IL‐17. AP‐1, Activator protein 1; C/EBP, CCAAT Enhancer Binding Protein; CD44, Cluster of Differentiation 44; ELK1, ETS Transcription Factor ELK1; ERK, Extracellular Signal‐Regulated Kinase; FOS, Fos Proto‐Oncogene; FOXC1, Forkhead Box C1; HDAC1, Histone Deacetylase 1; HIF‐1 α, Hypoxia Inducible Factor 1 Subunit Alpha; HMMR, Hyaluronan Mediated Motility Receptor; IL‐17, Interleukin 17; JNK, c‐Jun N‐terminal kinase; JUN, Jun Proto‐Oncogene; mTORC1, Mechanistic target of rapamycin complex 1; NF‐kB, Nuclear factor kappa B; p38, p38 MAPK; RORγt, Retinoic Acid‐related Orphan Receptor Gamma T; ROS, Reactive Oxygen Species; RUNX 1, RUNX Family Transcription Factor 1; S6K1, Ribosomal Protein S6 Kinase Beta‐1; S6K2, Ribosomal Protein S6 Kinase Beta‐2; STAT3, Signal Transducer And Activator Of Transcriptibon 3; TLE3, TLE family member 3.

What is more important, IL‐17 mediates inflammatory reactions via p38/c‐Fos, and JNK/c‐Jun activation was proved in an AP‐1‐dependent manner (including FOS),^61^ and blockade of interleukin 17 (IL‐17) or tumour necrosis factor alpha signalling in psoriasis following upregulation of FOXC1 was also demonstrated.^62^ In several malignancies, excessive activation of MAPK and PI3K signalling pathways leads to frequent induction of FOXC1 transcription by downstream transcription factors, including AP‐1 and NF‐κB.^63^ Therefore, it is easy to assume that there may be an upstream and downstream relationship between IL‐17, FOS and FOXC1 (Figure 11). Moreover, in cases of acute myelogenous leukaemia, FOXC1 knockdown induces the loss of repressor proteins, the gain of CCAAT Enhancer Binding Protein Alpha (CEBPA) binding and the upregulation of nearby genes.^64^ The C/EBP binding motif was highly conserved in the HMMR promoter region.^65^ In our work, there was a significant negative correlation between FOXC1 and HMMR (Figure 9A,B), and HMMR did not show a direct co‐expression relationship with FOS, FOSB and ATF3 (Figure 9D). We suggest that FOS and FOXC1, which IL17 downregulates in MASH and DN, may further affect HMMR expression. IL17/FOS/FOXC1/HMMR regulatory axis can potentially influence MASH and DN disease progression (Figure 11).

HMMR, a hyaluronan (HA)‐binding protein, manifests multiple functions with different cellular localizations. HMMR‐dependent ERK phosphorylation was observed in many cell types^66^ while decreasing ERK phosphorylation has been shown with HMMR knockdown.^67^ ERK phosphorylation can activate T‐helper 17 (Th17) cells, which can induce inflammatory diseases.^68^ By contrast, recent studies have shown that phosphorylation of serine 182 of RORγt by ERKs can limit excessive activation of Th17, inhibiting the expression of IL17.^69^ It is strongly suggested that intracellular HMMR plays a double‐edged role in participating in the production of IL17 (Figure 11).

Moreover, consistent with the enrichment of the IL‐17 signalling pathway, the PI3K‐Akt‐mTOR pathway (or ECM‐receptor interaction genes) was enriched in Figure 9D, and the two pathways could be bridged through the IL‐18 signalling pathway. Considering that some of the signalling pathways that crosstalk with the PI3K‐Akt‐mTOR pathway activated by HMMR have been reported, including EGFR/AKT/ERK,^70^ ROK‐PI3K^71^ and the evidence of mTOR‐SRF axis regulates HMMR expression in human prostate cancer cells.^72^ Therefore, we speculate that a regulatory relationship may exist between PI3K‐Akt‐mTOR and HMMR related to IL17. In addition, it is interesting to note that the positive correlation between HMMR and glycolysis is confirmed,^73^ and the glycolytic intermediate dihydroxyacetone phosphate (DHAP) relays glycolytic activity to mTORC1 signalling.^74^ mTORC1 was not only considered a nutrient and energy sensor responding to changes in growth factor, amino acid and nutrient levels in type 2 diabetes^75^ but also plays a critical role in IL‐17 expression.^76^ We, therefore, suggest that the HMMR may influence the IL‐17 signalling pathway through the PI3K‐Akt‐mTOR pathway (Figure 11).

Previous studies have emphasized that HMMR expression enhanced cell proliferation, the expression of mesenchymal markers and cell invasion in bladder cancer cells.^77^ In addition, HMMR is associated with immune infiltration and is a prognostic biomarker for lung adenocarcinoma.^45^ Our Immune infiltration analysis shows an accumulation of mast cells resting in both MASH and DN (Figure 10A,B). Several studies have confirmed that mast cells LAD2 cultured under hypoxia conditions exhibited significantly decreased expression of HMMR, and the cell's adhesion to hyaluronic acid (HA) decreased rapidly.^78^ Also, treating cells with hyaluronidase triggers a robust increase in glycolysis with high glucose uptake.^73^ Subsequently, the cells can upregulate glycolysis to limit reactive oxygen species (ROS) production and readily switch back to oxidative phosphorylation.^79^ Glucose can be diverted to the pentose phosphate pathway to generate NADPH, protecting cells from ROS as the main intracellular antioxidant and maintaining dynamic redox balance.^80^ Overexpression of FOXC1 might be induced by the ROS dependent on the extracellular regulated protein kinases 1 and 2 (ERK1/2)‐ phospho‐ETS Transcription Factor 1 (p‐ELK1) pathway.^81^ It should be added that while the ERKs phosphorylate TCF/Elk‐1 and induce c‐Fos synthesis, they do not phosphorylate c‐Jun or c‐Fos on sites that potentiate their transcriptional activities.^82^ Therefore, based on the transformation of glycolysis, FOS and FOXC1 has the potential to be downstream molecules of extracellular HMMR (Also includes intracellular HMMR). Also, the extracellular HMMR bind to HA might trigger the CD44 intracellular signal transduction,^83^, ^84^ initiating nuclear localization of NF‐κB (a transcript factor binding to FOXC1 promoter). For the resting mast cells continuously sample their microenvironment and receive signals from their microenvironment to modulate the intensity of their response,^85^ HMMR has the potential to become a medium for resting mast cells to acquire microenvironmental signals.

More importantly, mast cells can be a major source of IL‐17,^86^, ^87^ which may play key regulatory roles in host defence and inflammatory diseases.^88^, ^89^ In our work, AP‐1 transcription factors (FOS, FOSB and ATF3) and FOXC1 transcription factors were significantly positively correlated with the activation of the mast cell (Figure 10C,D), indicating that the producing of IL‐17 from mast cells is perhaps related to these transcription factors. Although in the hypothetical IL17/FOS/FOXC1/HMMR regulatory axis, HMMR is upregulated. Our results yet show no significant difference in the expression of IL‐17 (Tables S2 and S3), which aligns with our earlier speculation that intracellular HMMR plays a dual role in IL‐17 production (Figure 11). There may be another reason why IL‐17 is not elevated: upregulated HMMR may be secreted outside the cell. More interestingly, mast cells communicate with the microenvironment in the resting state through two elegant secretory mechanisms, piecemeal degranulation (PMD) and exosome release.^90^, ^91^ Therefore, we suggest that there is a possibility of HMMR being carried away by resting mast cell vesicle isolation. As the only ECM‐receptor interacting genes in PPI cluster 1 (Figure 6A), it is reasonable to believe that this mobile HMMR brings additional signals to mast cells, perhaps associated with the downregulation of AP‐1 transcription factors. Since HMMR release by exosomes has been confirmed,^92^ we propose that once the HMMR can be accessible to another tissue, it can affect target cells regarding transcriptional expression, cell differentiation, signalling and glucose metabolism.

In summary, based on the current research results, we can suggest three potential relationships between MASH and DN: (i) the biological processes represented by IL‐17, TNF and Apoptosis as signalling pathways are present in both MASH and DN. (ii) a FOS/FOXC1/HMMR regulatory axis in MASH and DN may be associated with mast cells acting IL‐17 signalling pathway. (iii) The IL‐17 pathway represented by FOS, FOSB may be regulated by HMMR through the ERK or PI3K‐Akt‐mTOR pathway. Therefore, we speculate that HMMR may act as the signalling carrier between MASH and DN. However, these are still speculations, and more experiments are needed to verify the exact mechanism.

### 4.1 Strengths and limitations

The shared DEGs between MASH and DN do not contain ECM‐receptor interacting genes. Previous studies might fail to observe the shared role of ECM‐receptor interaction genes on MASH and DN. By applying gene co‐expression, and community analysis of PPI networks, we have, for the first time, labelled 19 CP genes that can affect MASH and DN. Combining machine learning and immune infiltration analysis, we conclude that FOS, HMMR and FOXC1 may be critical for regulating the IL‐17 signalling pathway in mast cells. Our research opens a new avenue for developing treatments for MASH and DN‐related symptoms. However, our study is still limited. For instance, we have not yet determined how these 19 CP genes are involved in ECM sensing of energy metabolism via the PI3K‐Akt‐mTOR pathway, which affects cytokines to initiate inflammation in target organs. Additionally, we have not observed the levels of HMMR and FOS during the crosstalk between the ECM pathway and the inflammatory pathway. The expression levels of genes play a crucial role in cellular functioning. For instance, the expression levels of caspase‐1 and GSDMD alone can determine the mode of inflammasome‐induced cell death.^93^ Additionally, depending on their expression, specific proteins like RIP1 can bidirectionally activate different cell signalling pathways, such as apoptosis and necroptosis.^94^ Going forward, we will investigate the regulatory mechanism of these 19 CP genes through gene expression analysis.

## 5 CONCLUSION

The present study shows that crosstalk genes between MASH and DN may be regulated by ECM‐receptor interaction genes, supporting that ECM influences the inflammatory responses of MASH and DN. The transcription factors FOS and FOXC1 associated with HMMR are involved in the activation process of mast cells and subsequently affect the IL‐17 signalling pathway. Since all three genes are associated with inflammation, they may help assess the degree of interaction between the two diseases and serve as potential biomarkers to guide future research.

## AUTHOR CONTRIBUTIONS

**Chao Chen:** Conceptualization (lead); software (lead); supervision (lead); writing – original draft (lead). **Yuxi He:** Software (equal). **Ying Ni:** Validation (lead); visualization (lead). **Zhanming Tang:** Data curation (equal); resources (equal); software (equal). **Wensheng Zhang:** Funding acquisition (lead); project administration (lead); writing – review and editing (equal).

## FUNDING INFORMATION

This work was supported by the National Nature Science Foundation of China (81771152).

## CONFLICT OF INTEREST STATEMENT

The authors declare that they have no known competing financial interests or personal relationships that could have appeared to influence the work reported in this paper.

## Supporting information


Figures S1–S4

Table S1

Table S2

Table S3

Table S4

Table S5

Table S6

Table S7

Table S8

## Data Availability

Publicly available datasets were analysed in this study. This data can be found in GEO data repository and include the accession numbers: GSE164760, GSE89632, GSE96804, and GSE30122.
