# Supplementary material for: Identification of crosstalk genes relating to ECM‐receptor interaction genes in MASH and DN using bioinformatics and machine learning
Source: J Cell Mol Med. 2024 Mar 1;28(6):e18156. doi: 10.1111/jcmm.18156 (PMC10907849; doi:10.1111/jcmm.18156)
Supplement: Supplementary file 1 — Figures S1–S4 [file JCMM-28-e18156-s005.zip › JCMM_18156.docx]

Figure S1. Removing batch effects in the analysis of expression microarray data. (A) Removing the batch effect from the MASH group; (B) Removing the batch effect from the DN group.

Figure S2. Flow chart of this study design.

Figure S3. Correlation of ECM and crosstalk genes. (A-B) The heatmap of ECM-receptor interaction genes in MASH and DN; (C-D) Pearson correlation heatmap of ECM and crosstalk genes in MASH and DN.

Figure S4. CP genes ROC curves analysis. (A-D) The median gene expression content in the disease was more significant than in the control group; (E-H) The median gene expression content in the disease was lower than in the control group.
