# Supplementary figures and images for: Identification of crosstalk genes relating to ECM‐receptor interaction genes in MASH and DN using bioinformatics and machine learning
Source: J Cell Mol Med. 2024 Mar 1;28(6):e18156. doi: 10.1111/jcmm.18156 (PMC10907849; doi:10.1111/jcmm.18156)

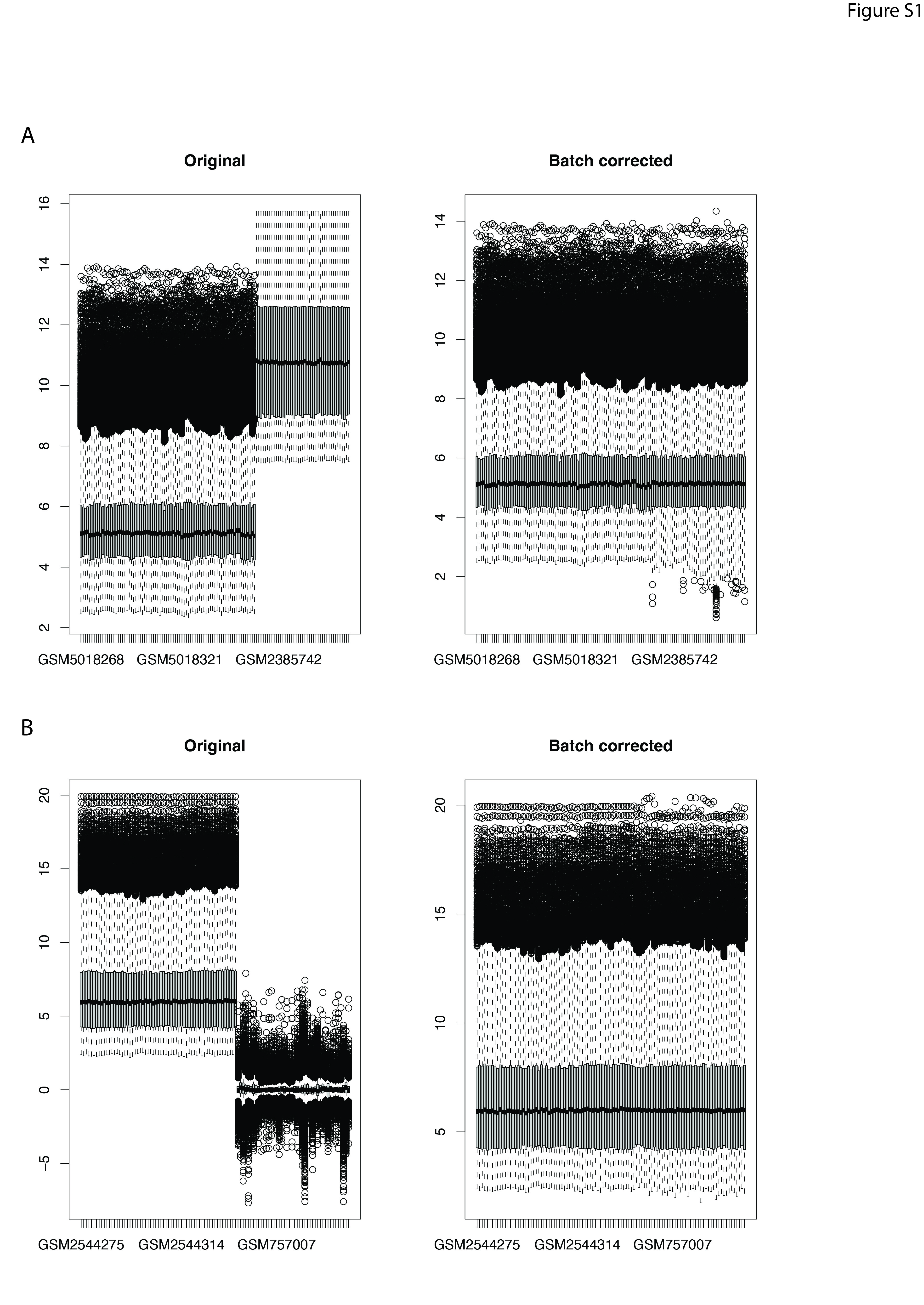

Supplement: Supplementary file 1 — Figures S1–S4 [file JCMM-28-e18156-s005.zip › jcmm18156-sup-0001-fig S1.jpg]

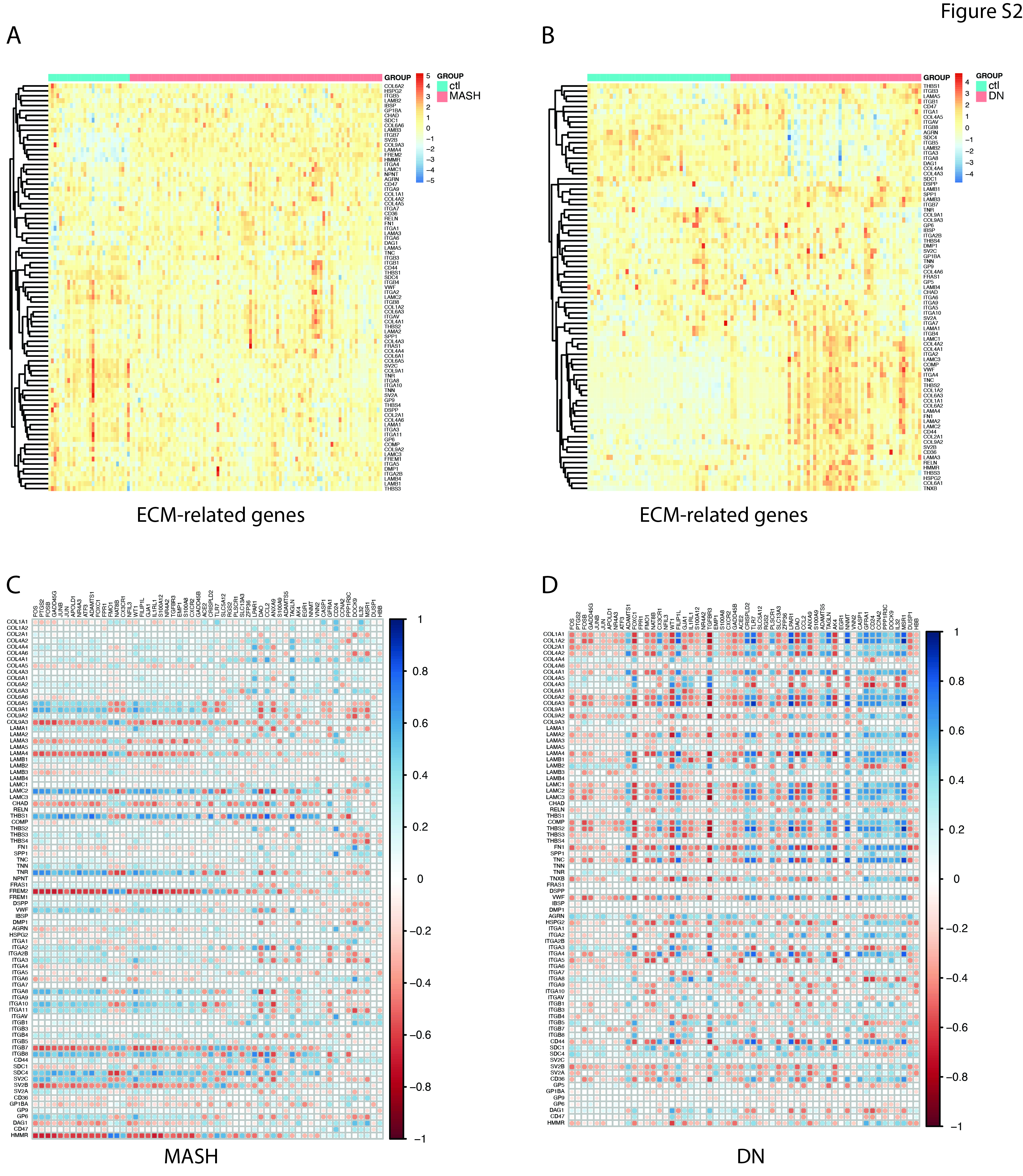

Supplement: Supplementary file 1 — Figures S1–S4 [file JCMM-28-e18156-s005.zip › jcmm18156-sup-0002-fig S2 fig4.jpg]

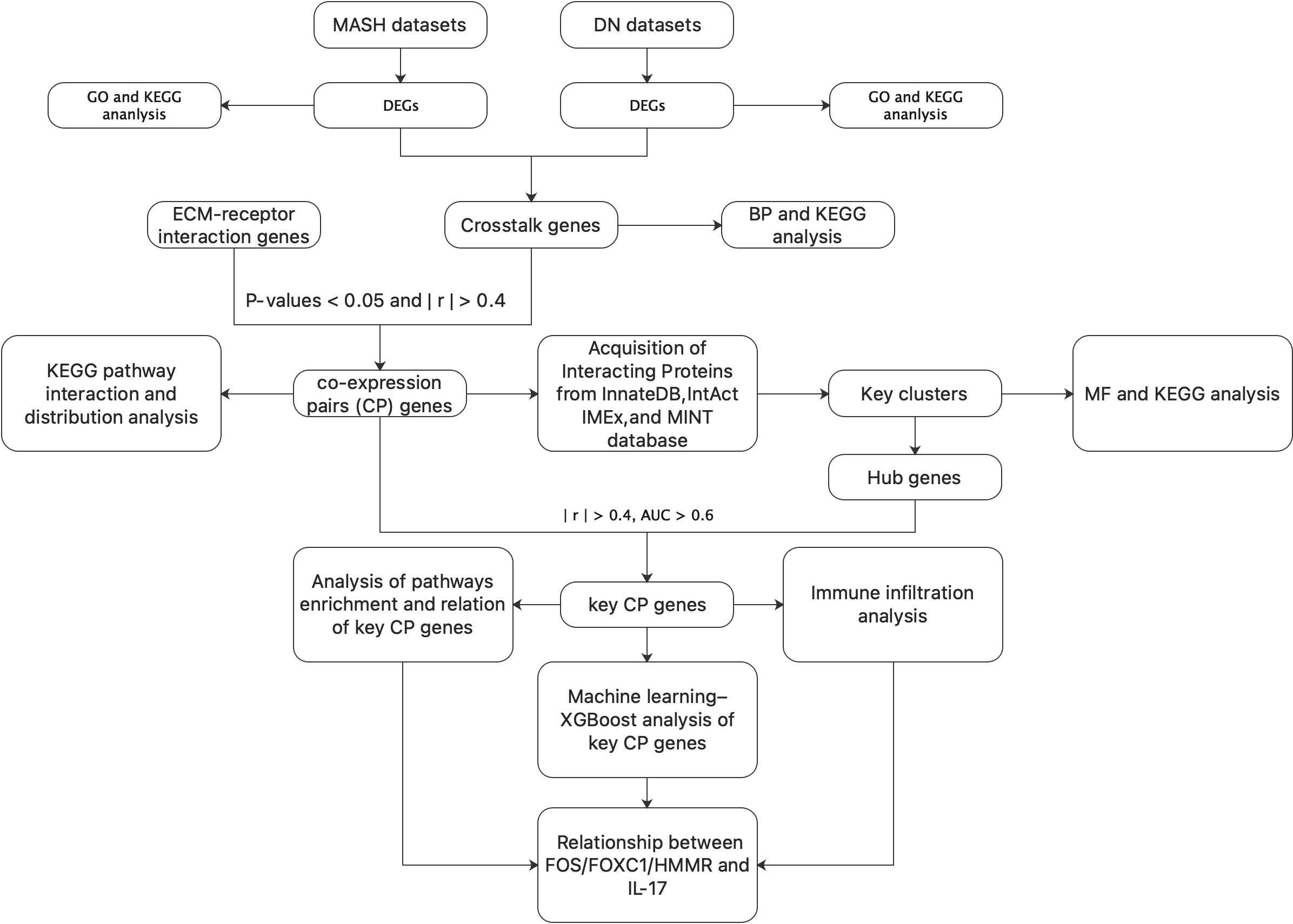

Supplement: Supplementary file 1 — Figures S1–S4 [file JCMM-28-e18156-s005.zip › jcmm18156-sup-0003-fig S3.jpg]

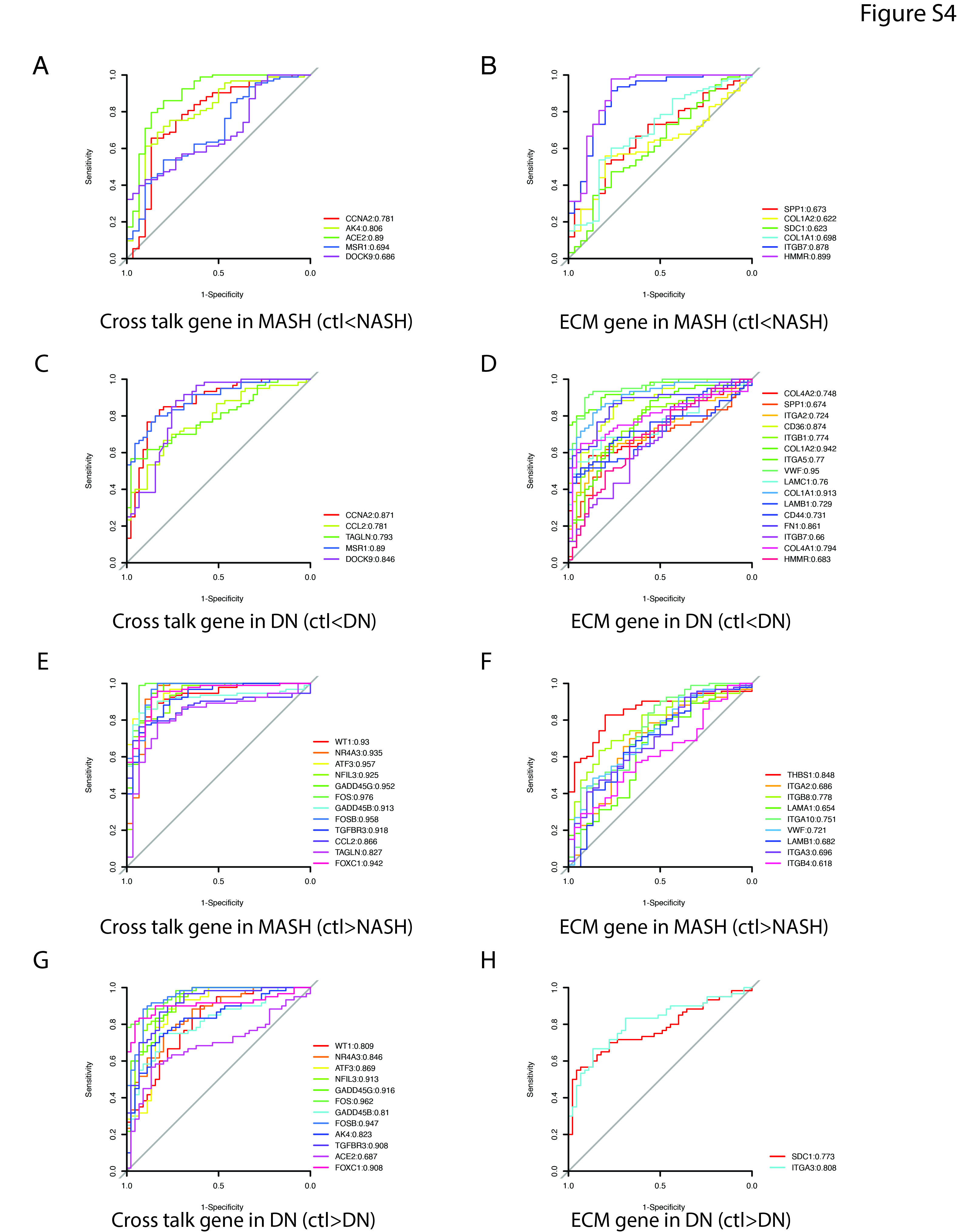

Supplement: Supplementary file 1 — Figures S1–S4 [file JCMM-28-e18156-s005.zip › jcmm18156-sup-0004-fig S4-7-8.jpg]
